# Supplementary material for: Rational Design of Novel Conjugated Terpolymers Based on Diketopyrrolopyrrole and Their Applications to Organic Thin-Film Transistors
Source: Polymers (Basel). 2023 Sep 18;15(18):3803. doi: 10.3390/polym15183803 (PMC10535888; doi:10.3390/polym15183803)

Supplementary Information

Figure S1. ^{1}H NMR spectra of Compound DPP-S-C8C10

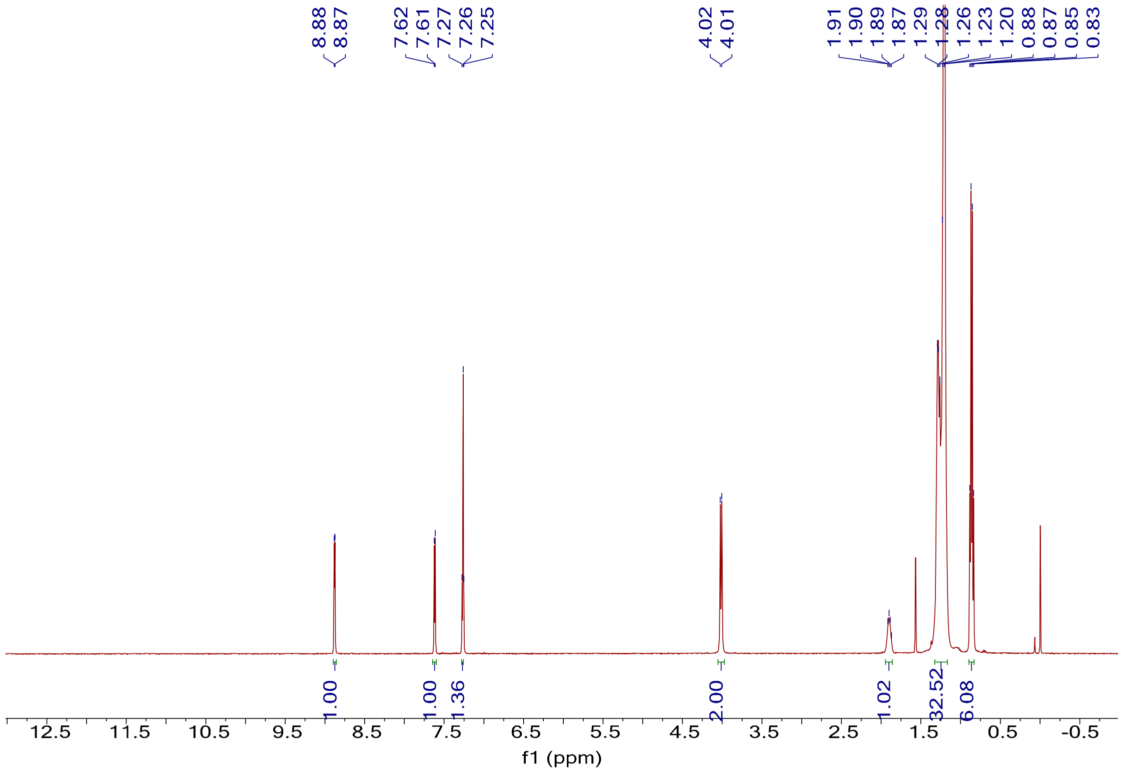

Figure S2. ^{1}C NMR spectra of Compound DPP-S-C8C10

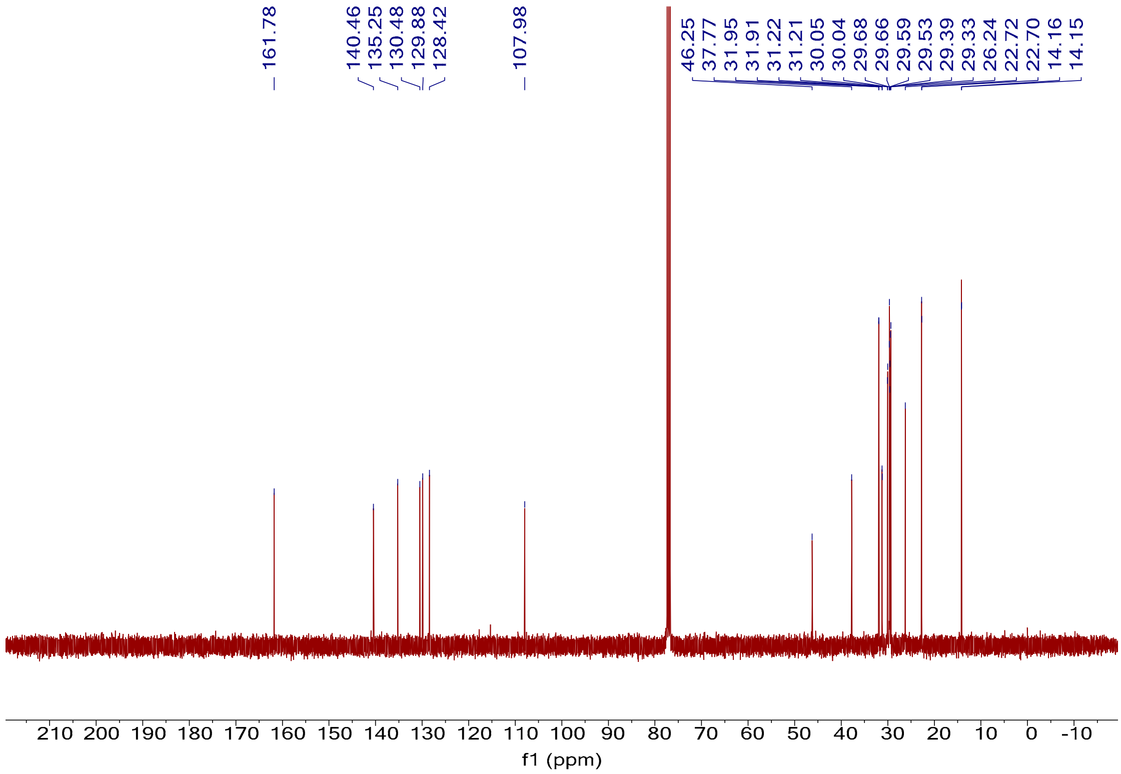

Figure S3. ^{1}H NMR spectra of Compound DPP-S-Br

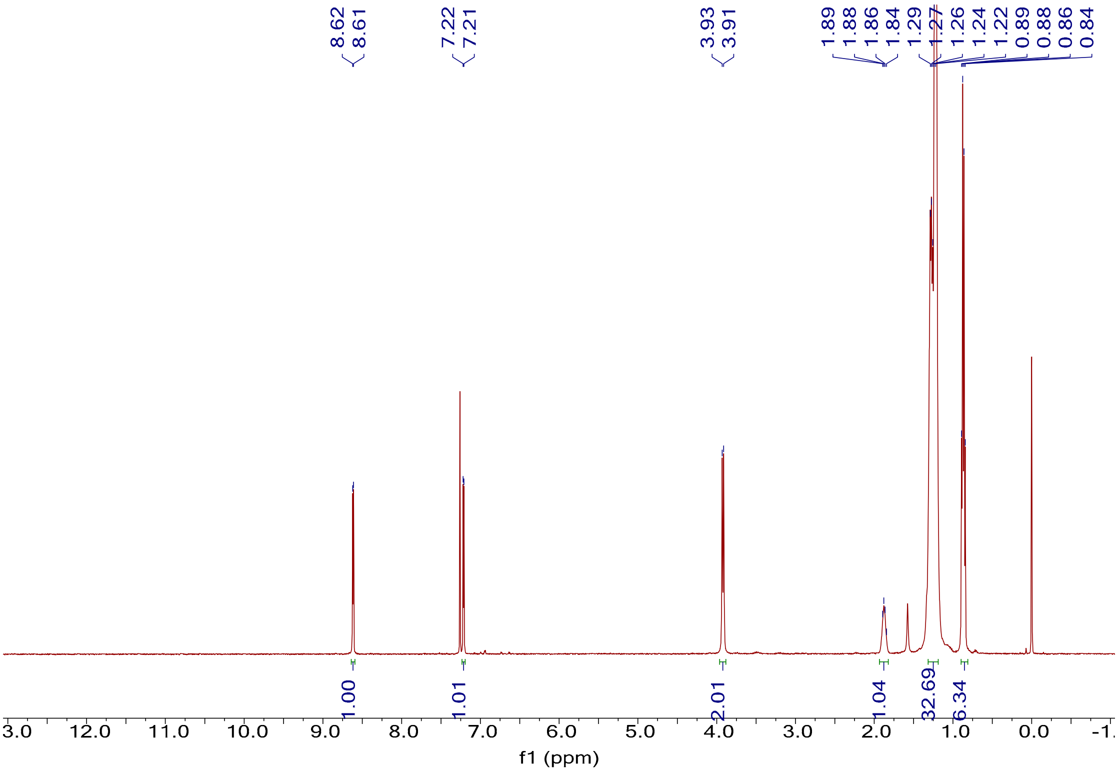

Figure S4. ^{1}H NMR spectra of Compound DPP-S-Br

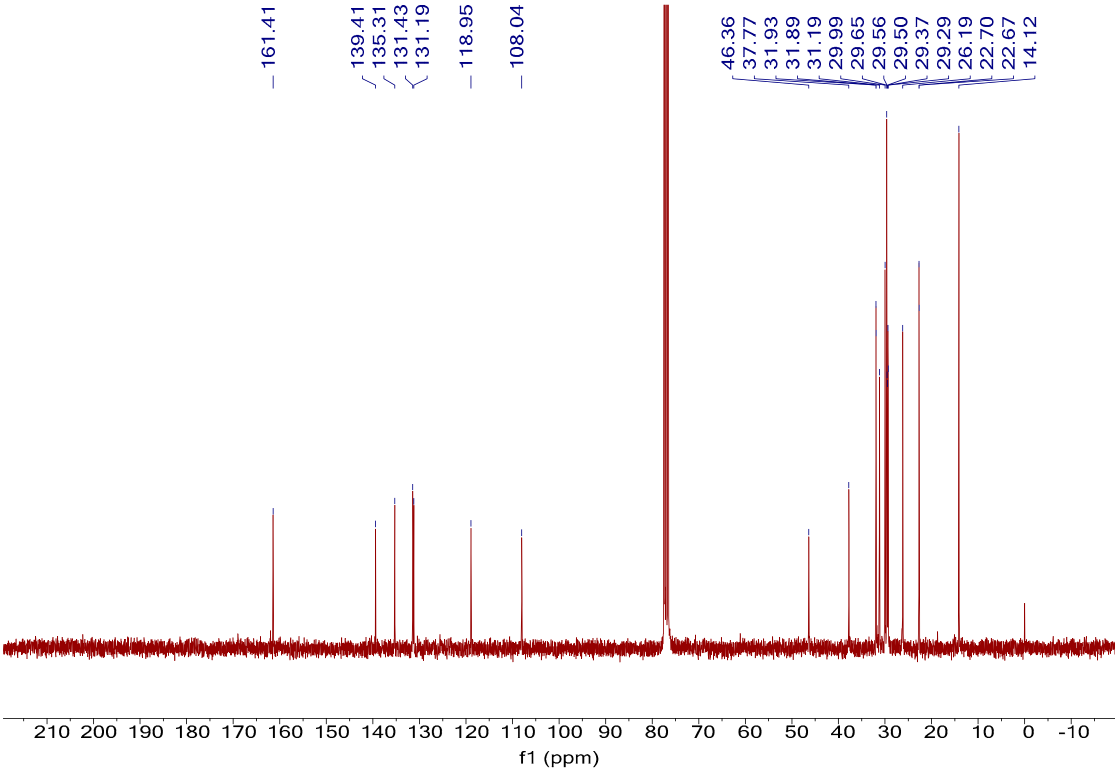

Figure S5. Mass spectra of Compound DPP-S-C8C10

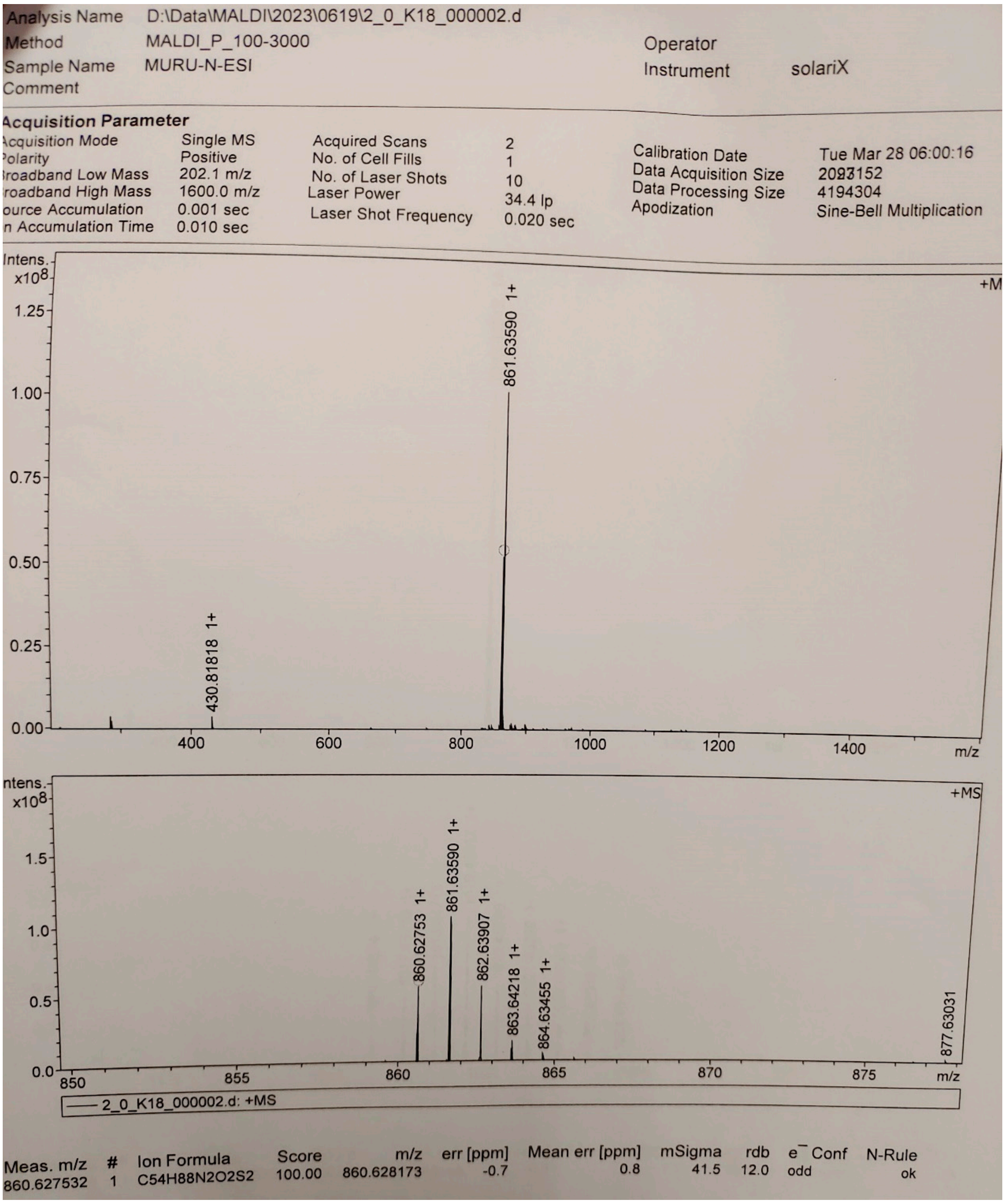

Figure S6. Mass spectra of Compound DPP-S-Br

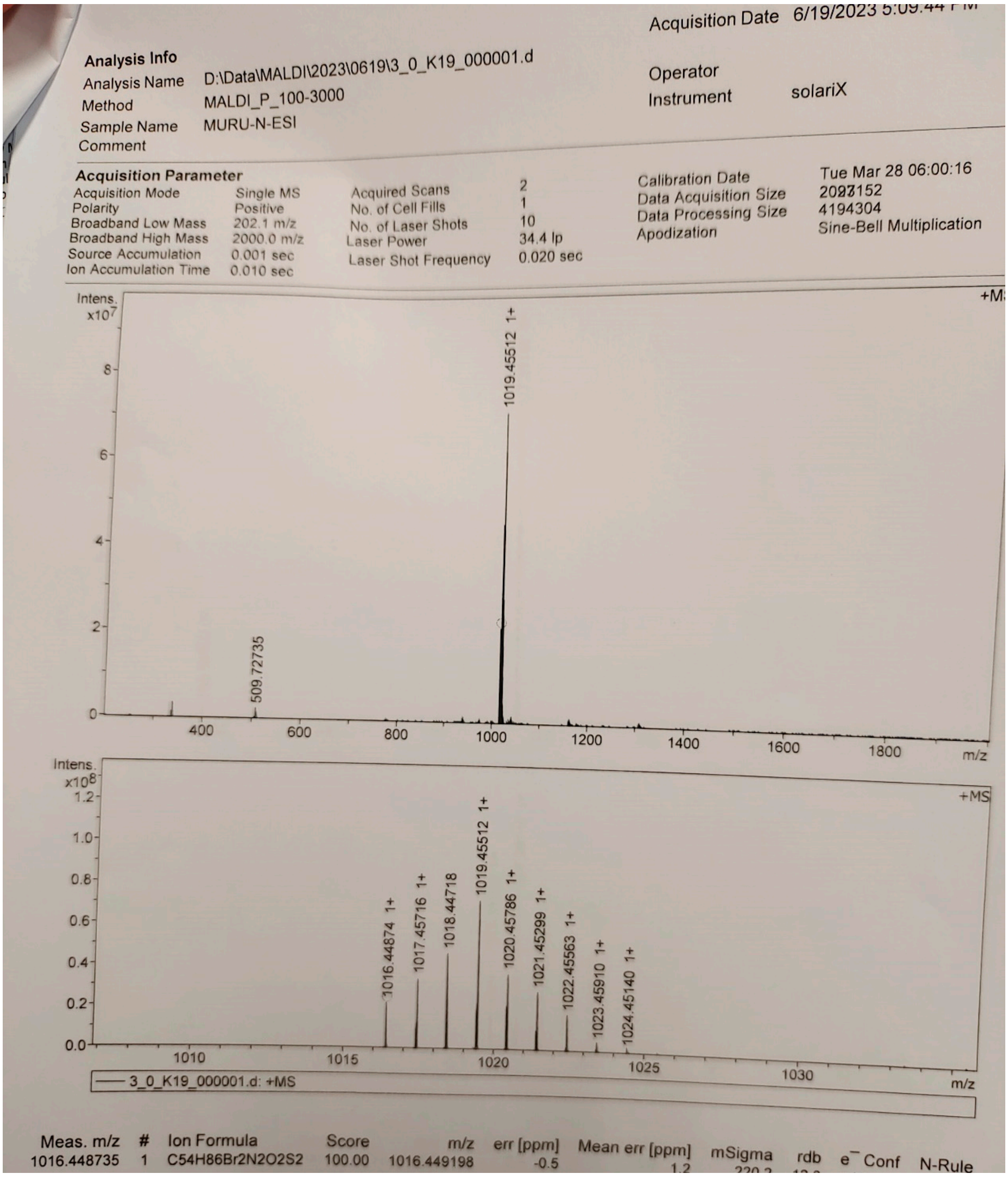

Figure S7. GPC data (cumulative percent curves and molecular weight distribution) for the four polymers.

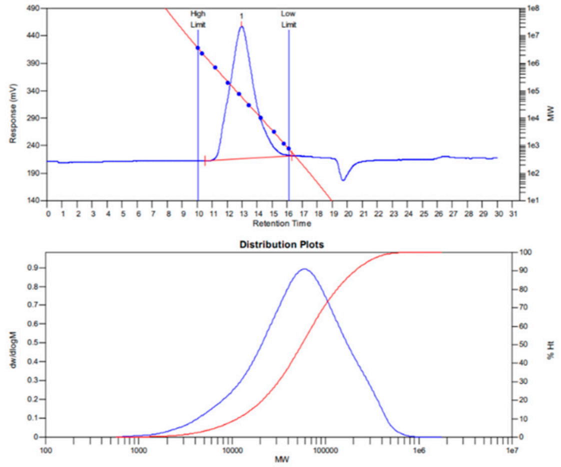

MW Averages

Peak No	Mp	Mn	Mw	Mz	Mz+1	Mv	PD
1	59675	24541	85717	185543	295065	74412	3.49281

Processed Peaks

Peak No	Name	Start RT (mins)	Max RT (mins)	End RT (mins)	Pk Height (mV)	% Height	Area (mV.secs)	% Area
1		10.55	12.97	16.32	240.706	0	27253.4	100

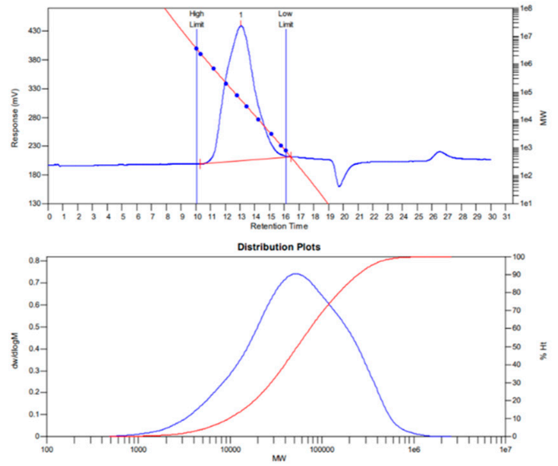

MW Averages

Peak No	Mp	Mn	Mw	Mz	Mz+1	Mv	PD
1	52040	21933	96864	249926	444756	81253	4.41636

Processed Peaks

Peak No	Name	Start RT (mins)	Max RT (mins)	End RT (mins)	Pk Height (mV)	% Height	Area (mV.secs)	% Area
1		10.28	13.07	16.43	234.623	0	31908.5	100

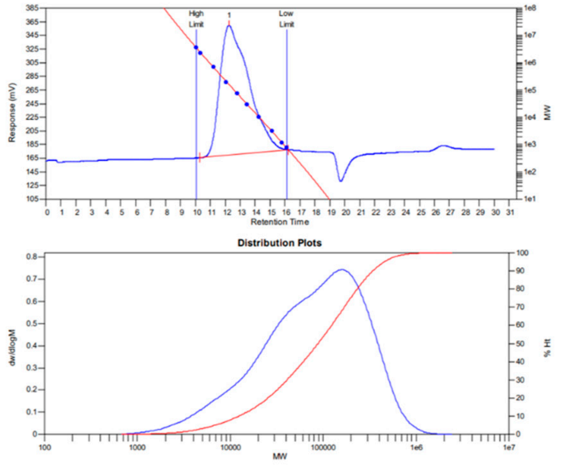

MW Averages

Peak No	Mp	Mn	Mw	Mz	Mz+1	Mv	PD
1	159731	28506	139502	313657	503861	118889	4.89378

Processed Peaks

Peak No	Name	Start RT (mins)	Max RT (mins)	End RT (mins)	Pk Height (mV)	% Height	Area (mV.secs)	% Area
1		10.32	12.25	16.20	189.911	0	25575.8	100

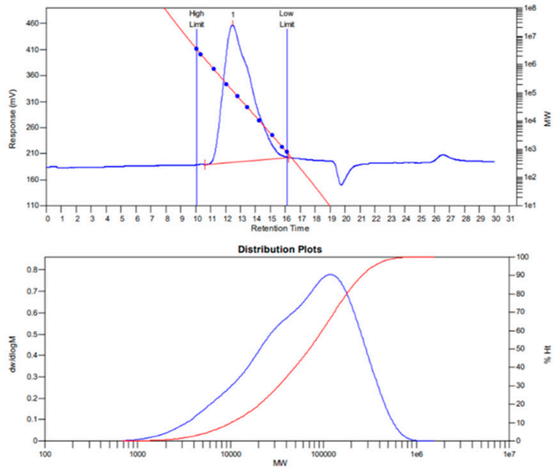

MW Averages

Peak No	Mp	Mn	Mw	Mz	Mz+1	Mv	PD
1	118513	24390	107112	227687	340905	92116	4.39164

Processed Peaks

Peak No	Name	Start RT (mins)	Max RT (mins)	End RT (mins)	Pk Height (mV)	% Height	Area (mV.secs)	% Area
1		10.63	12.47	16.20	263.388	0	33983.6	100

Figure S8. ^1H NMR spectra of Polymer Tz 0 %.

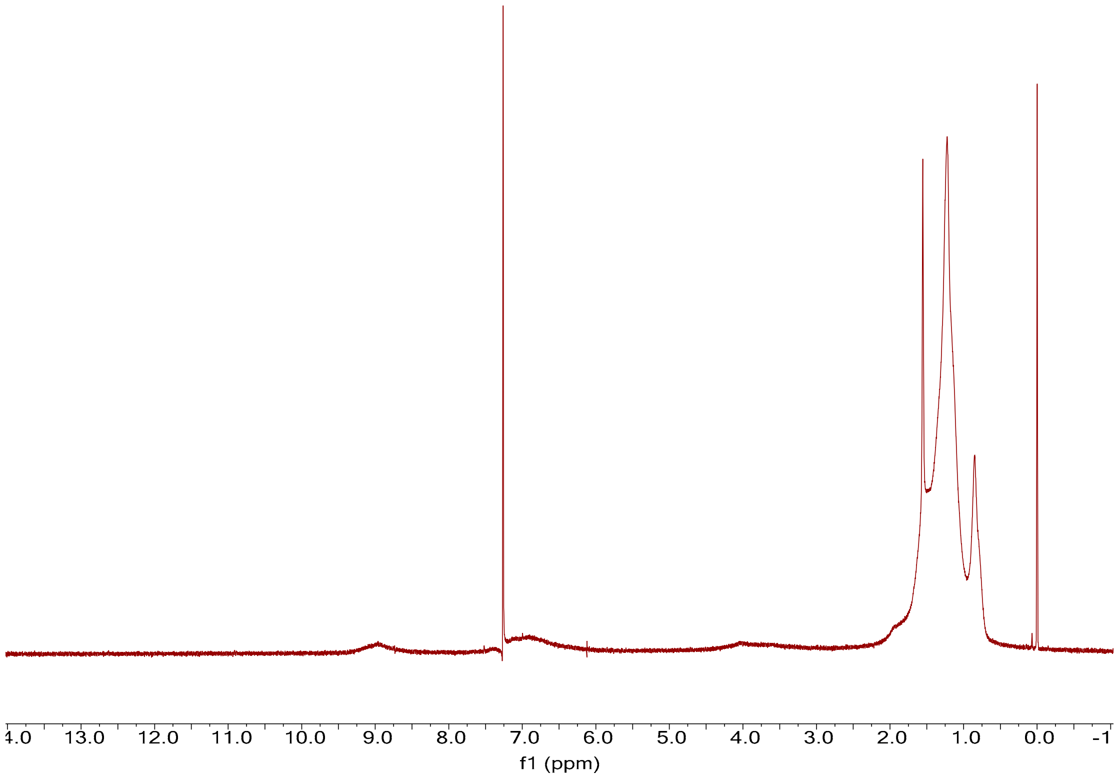

Figure S9. ^1H NMR spectra of Polymer Tz 25 %.

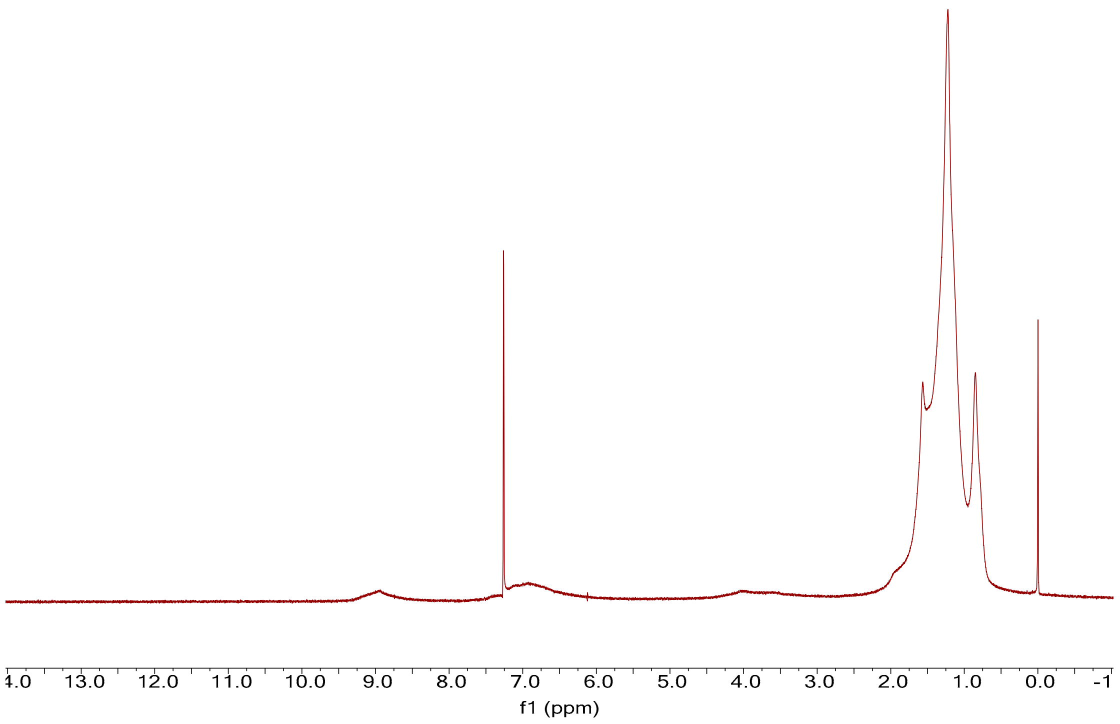

Figure S10. ^{1}H NMR spectra of Polymer Tz 50 %.

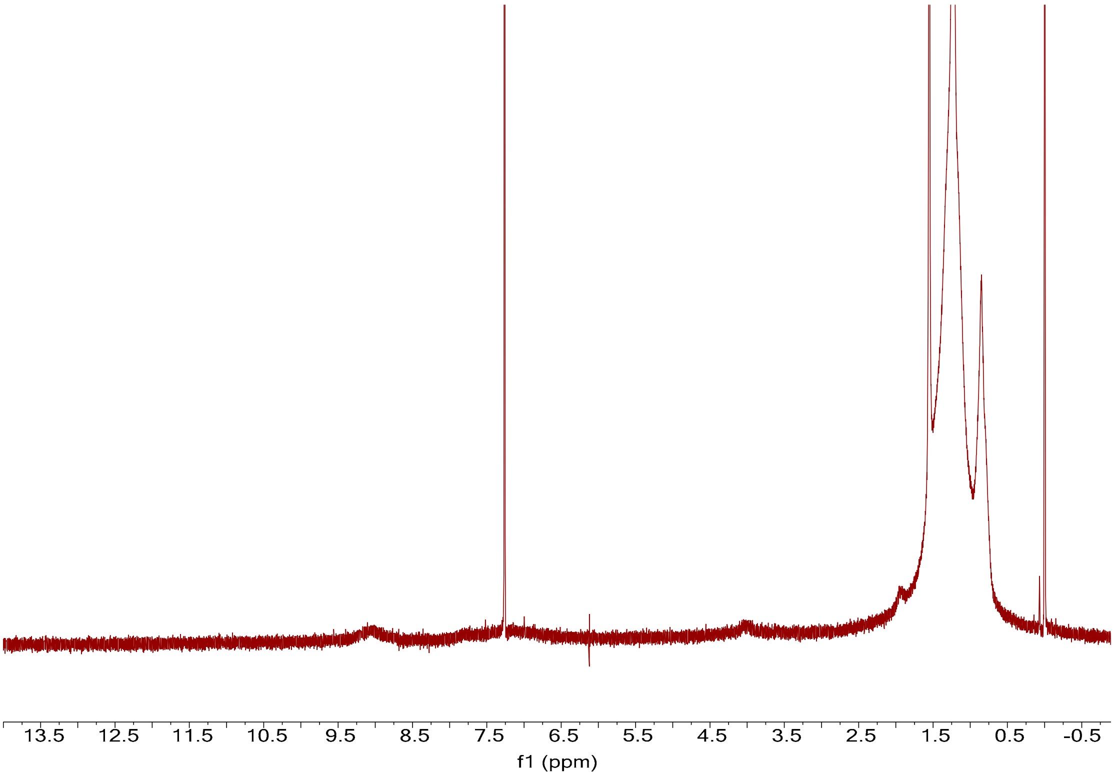

Figure S11. ^{1}H NMR spectra of Polymer Tz 75 %.

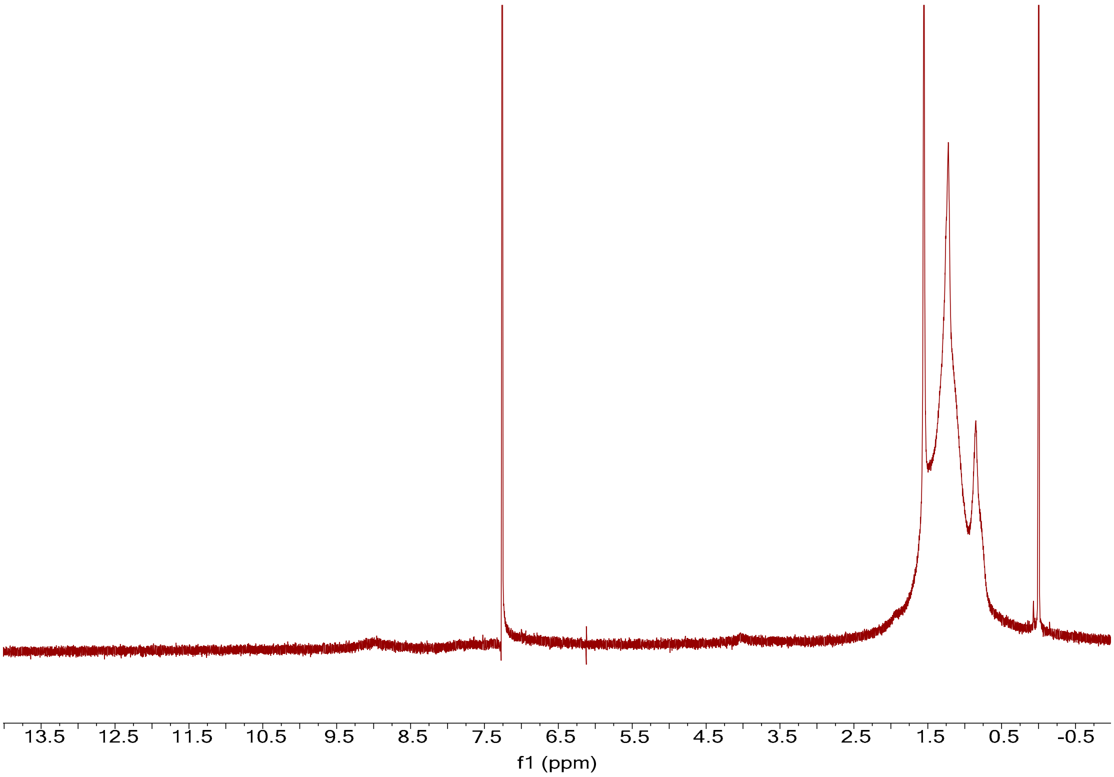

Figure S12. Solid-state ^{13}C NMR spectra of Polymer Tz 0 %.

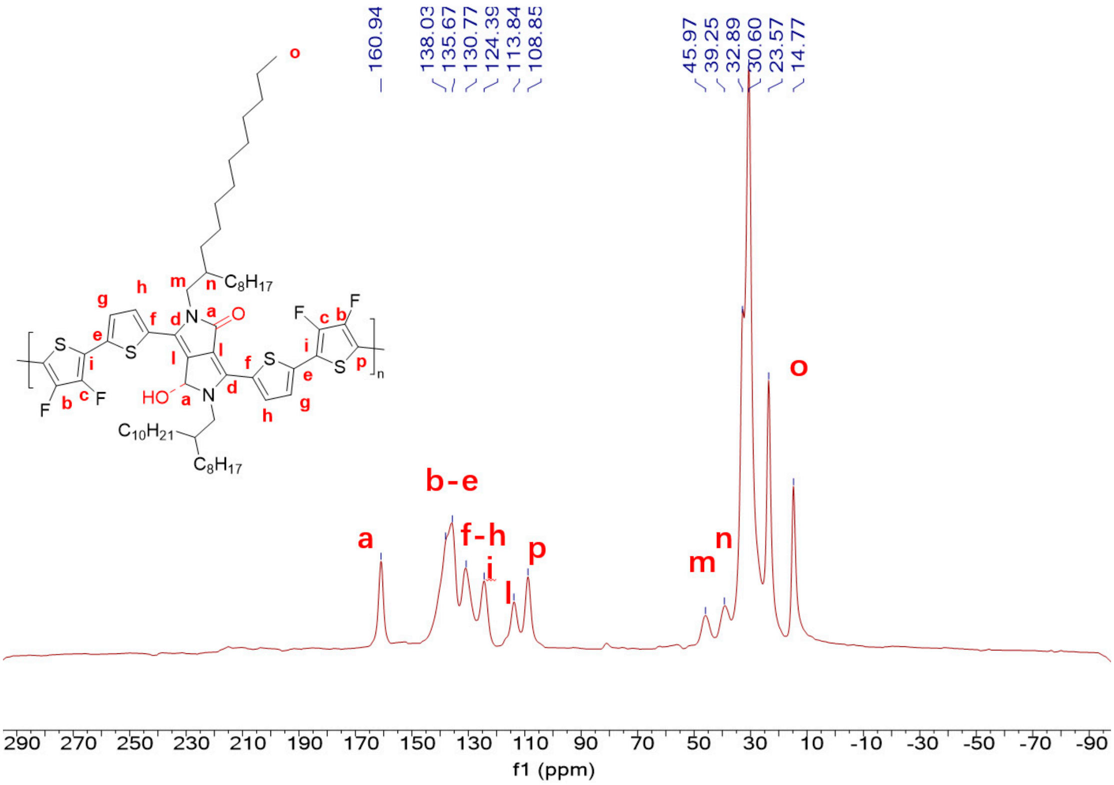

Figure S13. Solid-state ^{13}C NMR spectra of Polymer Tz 25 %.

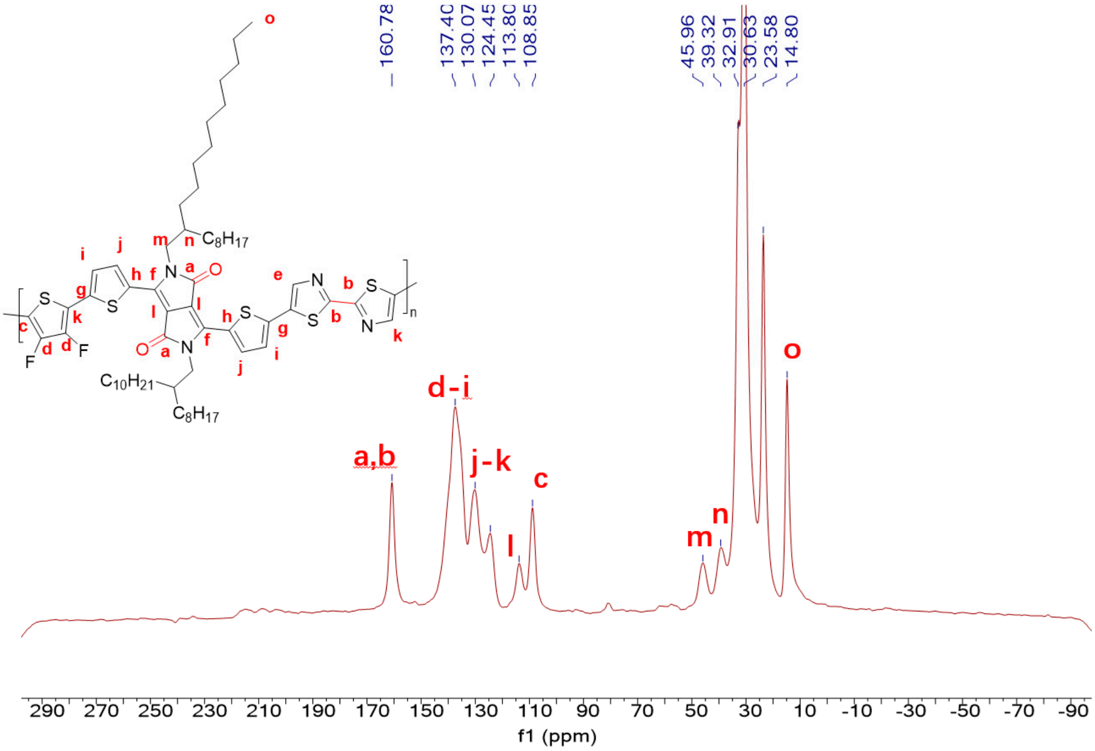

Figure S14. Solid-state ^{13}C NMR spectra of Polymer Tz 50 %.

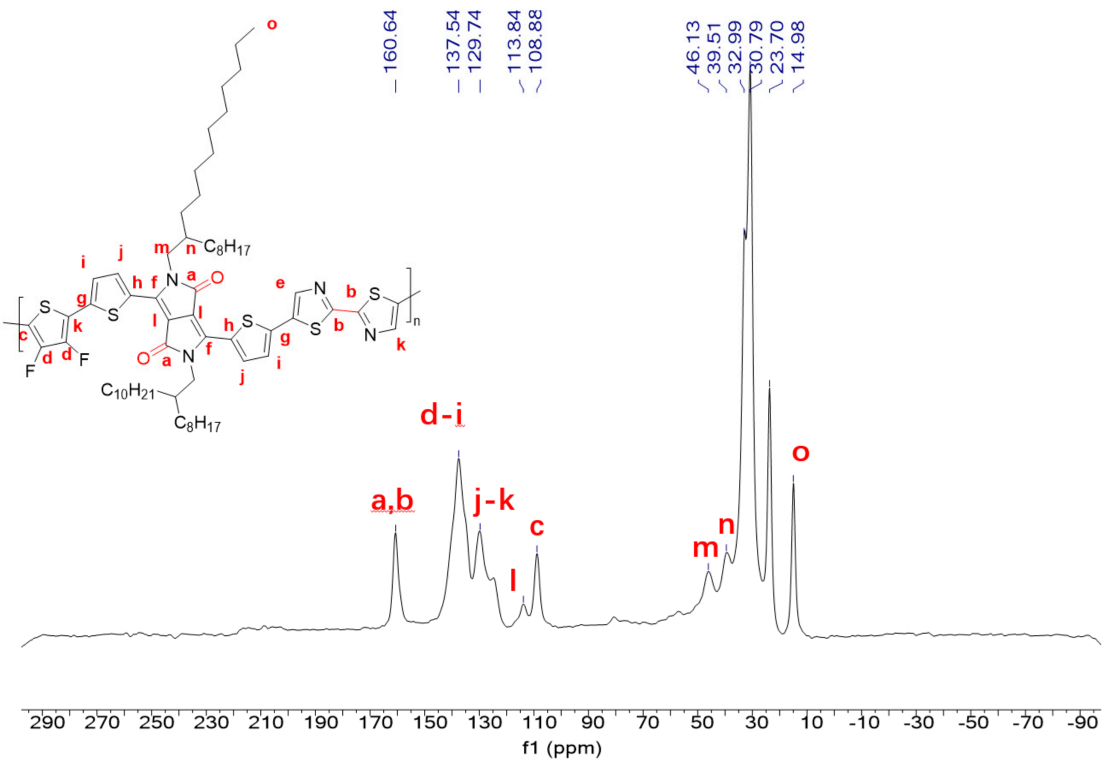

Figure S15. Solid-state ^{13}C NMR spectra of Polymer Tz 75 %.

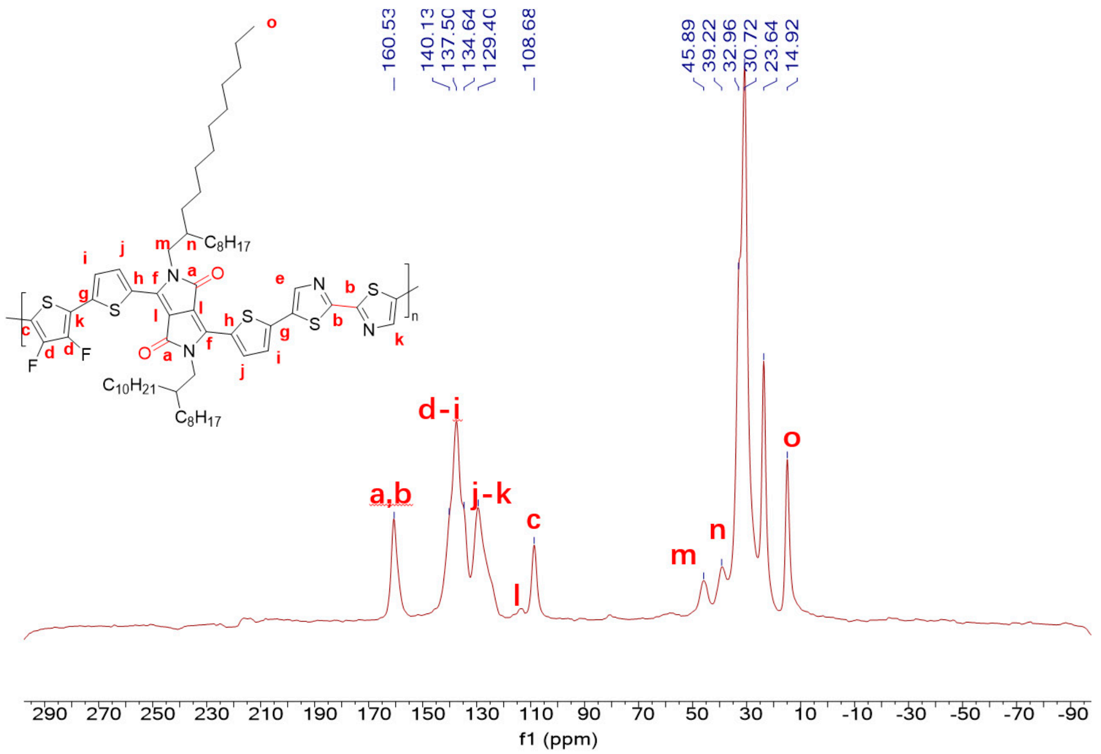

Figure S16. FT-IR spectra of the four polymers.

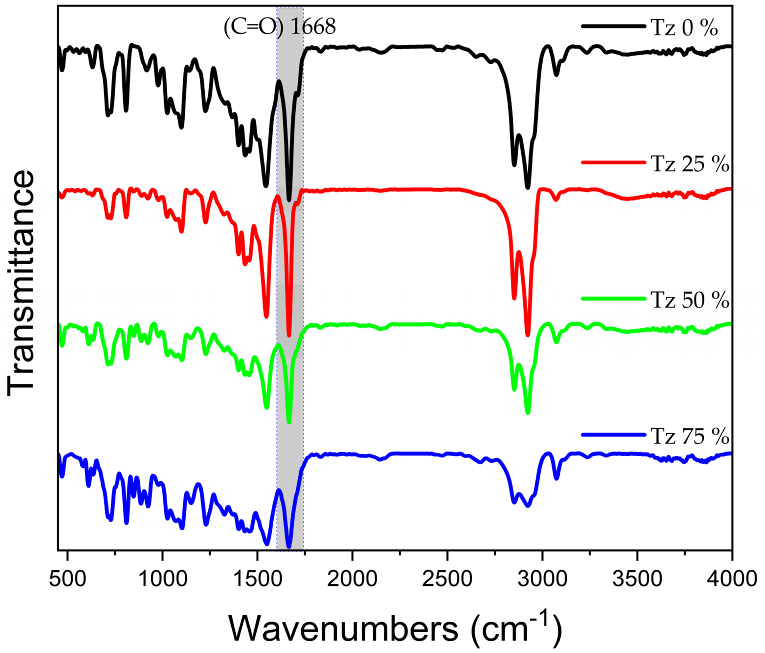

Figure S17. TGA analysis for the four polymers.

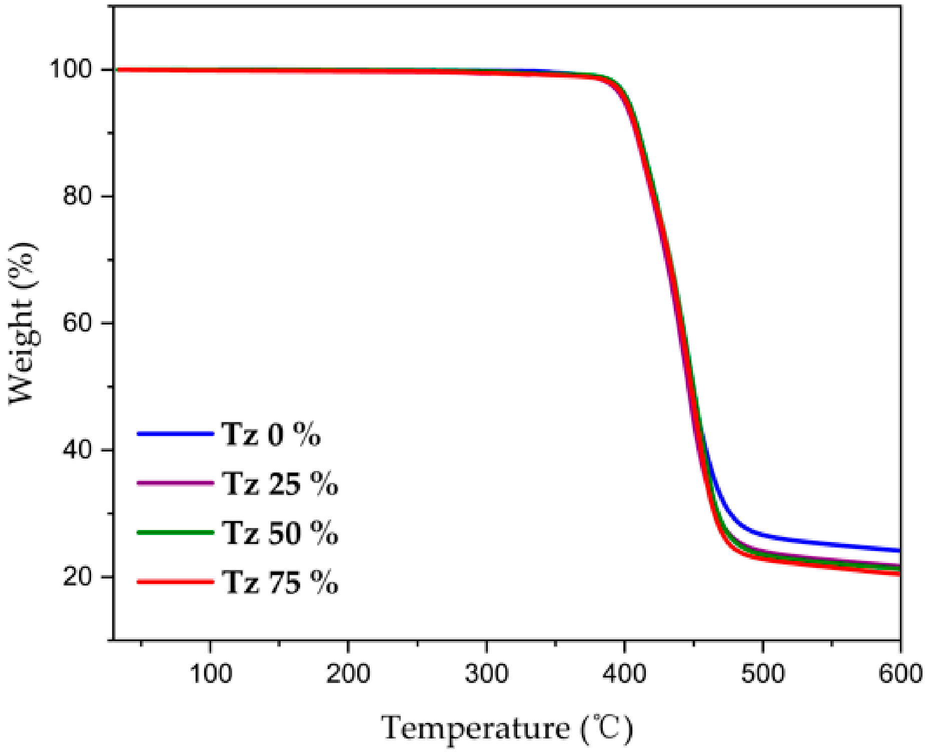

Figure S18. Normalized UV-vis spectrum of four polymers in chloroform solutions (left); in annealed thin films (right).

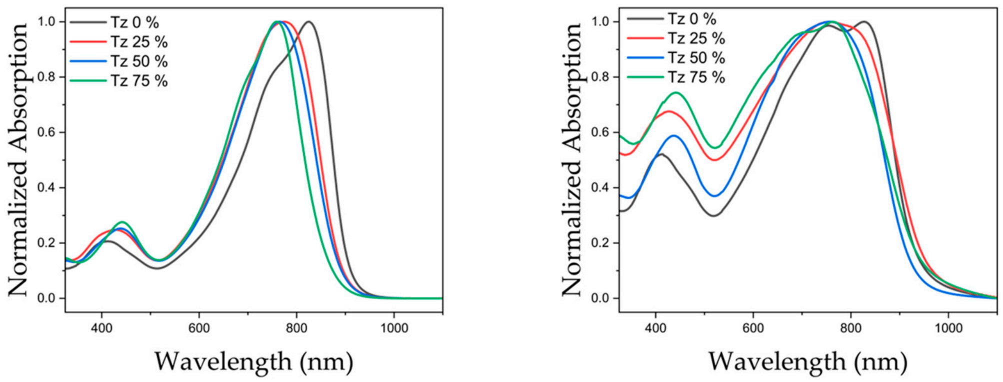

Table S1. UV-vis absorption of four polymers.

	λ_{max} (nm) [1]	λ_{max} (nm) [1]	λ_{max} (nm) [2]	λ_{max} (nm) [2]
Tz 0 %	826	411	830	412
Tz 25 %	776	420	762	426
Tz 50 %	765	442	756	439
Tz 75 %	758	444	754	443

[1] In solution; [2] In film;

Figure S19. Theoretical simulations of **(a)** Electrostatic potential surfaces and (**b**) Non-covalent interaction scattering of the dimer; **(c-d)** UV-vis spectrum of the dimer of the polymer calcuated by DFT at B3LYP/6-31G(d).

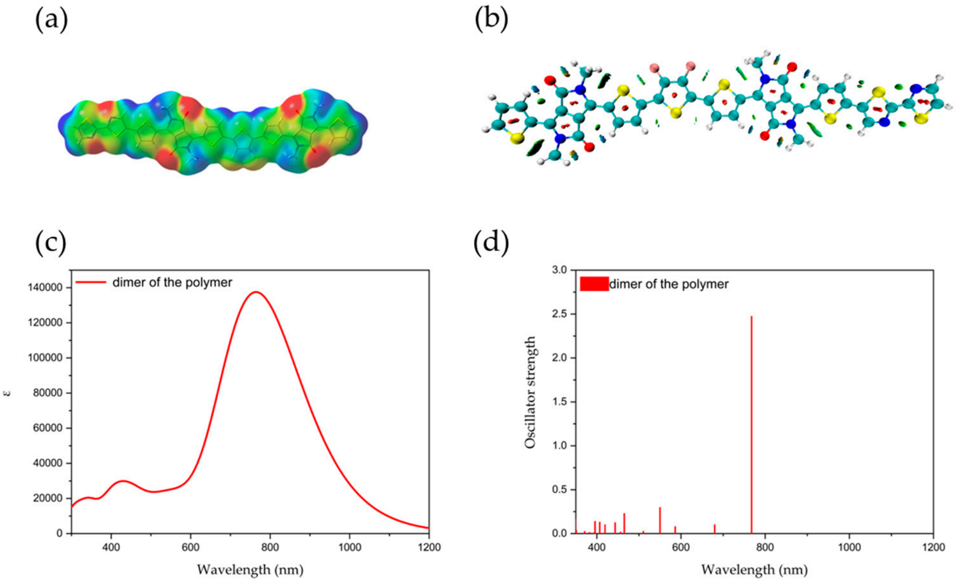

Figure S20. AFM height images of polymers annealed films of (a) Tz 0 %; (b) Tz 25 %; (c) Tz 50 %; (d) Tz 75 %.

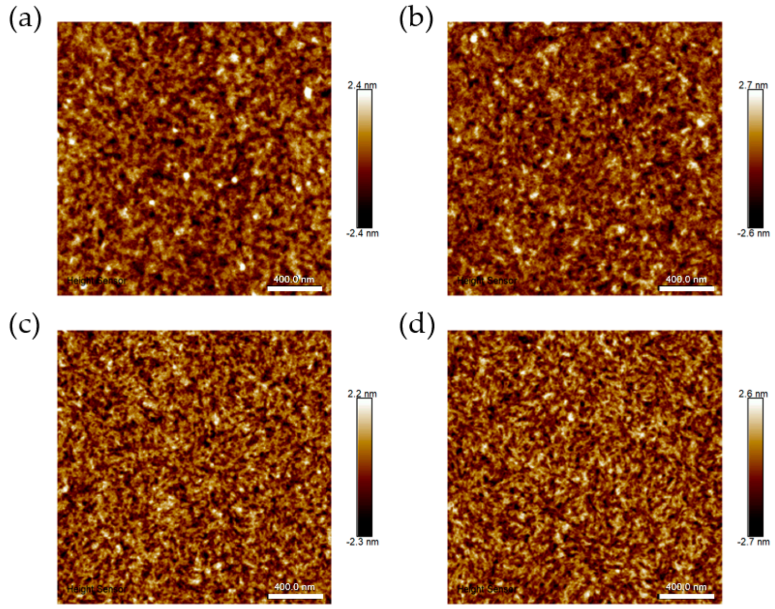

Supplement: Supplementary file 1 [file polymers-15-03803-s001.zip › polymers-2595466-supplementary.pdf]
